# Unmasking counterfeit semaglutide: analysis of real-world safety data from EudraVigilance

**DOI:** 10.3389/fphar.2026.1805842

**Published:** 2026-04-29

**Authors:** Alessia Zinzi, Mario Gaio, Rosanna Ruggiero, Annamaria Mascolo, Maria Antonietta Riemma, Mattia Cipriani, Ludovica Vittoria Laino, Liberato Berrino, Francesco Rossi, Annalisa Capuano

**Affiliations:** 1 Department of Life Science, Health, and Health Professions, Link Campus University, Rome, Italy; 2 Campania Regional Centre for Pharmacovigilance and Pharmacoepidemiology, Naples, Italy; 3 Department of Experimental Medicine – Section of Pharmacology “L. Donatelli”, University of Campania “Luigi Vanvitelli”, Naples, Italy

**Keywords:** counterfeit semaglutide, counterfeiting, eudravigilance, GLP-1, obesity, pharmacovigilance, safety

## Abstract

**Introduction:**

The spread of counterfeit drugs represents a serious threat to public health because they may be ineffective or cause the onset of severe suspected adverse drug reactions (ADRs). To date, the traceability of semaglutide-based products, widely used off-label for weight loss, is not a well-studied area.

**Methods:**

The aim of this study was to perform a descriptive analysis to explore and describe the characteristics of safety cases related to potential counterfeit semaglutide products by using the European pharmacovigilance database EudraVigilance. Individual Case Safety Reports (ICSRs) were retrieved over the period from 1 January 2018 to 31 December 2025.

**Results:**

A total of 234 Individual Case Safety Reports (ICSRs) related to potential counterfeit semaglutide were retrieved from EudraVigilance, of which N = 172 (73.5%) were related to females and N = 83 (35.5%) to adult patients. A total of 89.3% of all suspected ADRs were serious. The most frequently reported suspected ADRs were “vomiting”, “nausea”, and “hypoglycemia”. The analysis of disproportionality showed a higher reporting frequency of “hypoglycemia”, “product use in unapproved indication”, “malaise”, and “drug ineffective” with potential counterfeit semaglutide compared to non-counterfeit semaglutide.

**Discussion:**

The present pharmacovigilance study showed a useful role in identifying potential counterfeit medicine suspected ADRs. Further studies are warranted.

## Highlights

- The spread of counterfeit semaglutide poses a significant threat to public health.
- Most events related to potential counterfeit semaglutide concern female and adult patients.
- Potential counterfeit semaglutide showed an increased probability of reporting hypoglycemia, product use in unapproved indication, malaise, and ineffective drug.
- Continuous pharmacovigilance is essential to monitor and mitigate risks associated with counterfeit semaglutide.

## 1 Introduction

The strategies for the management of overweight or obesity have undergone significant evolution in recent years due to the introduction of innovative therapies. Semaglutide, a glucagon-like peptide-1 receptor agonist (GLP-1 RA), initially approved for the treatment of type 2 diabetes mellitus (T2DM), has emerged as an effective treatment for managing patients with obesity or overweight with at least one weight-related comorbidity (EMA, 2025; Prasad-Reddy and Isaacs, 2015; Lau et al., 2015). Semaglutide reduces body weight and fat mass by decreasing appetite and slowing gastric emptying in the postprandial phase (Sorli et al., 2017). In 2017, the SUSTAIN study (Semaglutide Unabated Sustainability in Treatment of Type 2 Diabetes) demonstrated that, in patients with type 2 diabetes, semaglutide improves body weight and glycated hemoglobin (HbA1c) levels (Davies et al., 2021). Subsequently, the results of the STEP 2 (Semaglutide Treatment Effect in People With Obesity) trial showed that among patients with overweight and obesity (without diabetes), once-weekly semaglutide 2.4 mg was associated with a weight reduction of up to 10.6% (Rodbard et al., 2019). Finally, in the PIONEER studies, oral semaglutide showed a significant improvement in HbA1c and greater weight reduction compared to empagliflozin (PIONEER 2), sitagliptin (PIONEER 3 and PIONEER 7), and liraglutide (PIONEER 4) (Rosenstock et al., 2019; Pratley et al., 2019; Pieber et al., 2019; Carboni et al., 2024). These important results have generated great interest among patients with obesity and their clinicians. Semaglutide use has also become widespread among individuals without the clinical characteristics indicated in the marketing authorization, eager to lose weight more easily and efficiently, and further encouraged to use semaglutide off-label by social media and celebrities (Suran, 2023). This has led to a shortage of supplies worldwide and complicated the treatment of diabetic patients being treated with this drug (Suran, 2023; Ashraf et al., 2024a). In this scenario, the growing global demand and the unavailability of semaglutide have fostered a thriving illicit market, often facilitated by the web, where it is possible to obtain semaglutide without a prescription, sometimes in counterfeit, low-quality, and poorly tolerated versions (Ashraf et al., 2024b; European Medicines Agency, 2015). Specifically, there are online shops that illegally direct users to purchase illicit drug products, where half of the transactions are fraudulent, and sellers are often involved in non-delivery scams in e-commerce, substandard or counterfeit products, and not manufactured according to current regulations (European Medicines Agency, 2015). Fortunately, Regulatory Authorities (World Health Organization and the European Medicines Agency) have identified and warned consumers of the presence of counterfeit semaglutide containing insulin or other potentially toxic substances responsible for adverse drug reactions (ADRs), including lack of drug efficacy (NABP, 2023; Food and Drug Administration, 2025). Recent case reports have also described serious clinical consequences associated with counterfeit semaglutide, such as euglycemic ketoacidosis following its use for weight loss (Sterckx and De Keyser, 2026). In this scenario, we decided to conduct a pharmacovigilance study focusing on potential cases of counterfeit semaglutide through the analysis of the European spontaneous reporting system database, EudraVigilance (EV).

## 2 Methods

### 2.1 Data source

Data on Individual Case Safety Reports (ICSRs) of suspected adverse drug reactions (ADRs) associated with semaglutide were retrieved from the EV database (EudraVigilance, 2025). We selected all ICSRs reported in EV from 1 January 2018, the year of the first semaglutide-related ICSR, to 31 December 2025. A data cleaning process was performed prior to analysis. Duplicate reports were identified and removed by comparing key variables, including the unique case identifier, patient demographic characteristics (age group and sex), reporting date, and reported suspected ADRs. When multiple ICSRs referred to the same case, only the most complete and recent version was retained, in accordance with standard pharmacovigilance practices. After deduplication, we considered the following information from each ICSR: a unique identifier number, the report type (spontaneous or non-spontaneous), the gateway receipt date, the primary source qualification (healthcare or non-healthcare professional), the primary source country (European or non-European Economic Area), the patient age group, the patient sex, and a list of suspected ADRs (type and seriousness). Seriousness was scored according to the International Council for International Organizations of Medical Sciences (CIOMS) criteria. Specifically, the suspected ADRs were considered serious if they led to (prolonged) hospitalization, were life-threatening events, resulted in death, disability, or congenital anomalies, or were classified as other medically important conditions (ESS, 2025). The latter represents a regulatory category that includes events which may not be immediately life-threatening or result in hospitalization but are considered clinically relevant and may require intervention to prevent serious outcomes. The reported suspected ADRs are coded as Preferred Term (PT) according to the Medical Dictionary for Regulatory Activities (MedDRA) (English, 2025). Each PT is a distinct medical concept for a symptom, sign, disease diagnosis, therapeutic indication, investigation, surgical or medical procedure, and medical social or family history characteristic. These data are publicly available on the European Medicine Agency (EMA) website (www.adrreports.eu, accessed on 05 January 2026) and have already been described as a valid system for pharmacovigilance studies (Rafaniello et al., 2020).

### 2.2 Study population

We included only ICSRs involving patients who used a potential counterfeit semaglutide-based product. To identify these cases, we relied on a list of PTs validated in a previous study (Pozsgai et al., 2022). Specifically, we selected ICSRs that reported at least one of the following PTs among the suspected ADRs: “counterfeit product administered”, “product counterfeit”, “product label counterfeit”, “product packaging counterfeit”, “suspected counterfeit product”, “adulterated product”, “product tampering”, and “suspected product tampering” (Pozsgai et al., 2022). The selection of ICSRs was based on the presence of these predefined MedDRA Preferred Terms and was therefore performed automatically. No additional manual case-by-case verification was conducted. These PTs are part of the MedDRA terminology and are assigned during the coding process of ICSRs within the pharmacovigilance system, based on the information provided by the original reporter. It is important to note that these PTs reflect reporter suspicion and do not provide confirmation that the product was actually counterfeit. Therefore, the identified cases should be interpreted as suspected counterfeit products rather than analytically confirmed falsified medicines.

As specified in the Data Source section, ICSRs were retrieved from the publicly accessible ADRreports.eu portal. The data extraction was initially filtered by selecting “semaglutide” as the active substance in the query interface. Therefore, all downloaded ICSRs already included semaglutide among the suspected medicinal products, regardless of the specific brand name or pharmaceutical formulation reported.

### 2.3 Descriptive analysis

We conducted a descriptive analysis of the demographic characteristics of the ICSRs and the main features of the reported suspected ADRs. Specifically, we examined the number of cases involving potential counterfeit semaglutide-based products, the sex and age of the involved patients, the geographical origin of the reports, and the reporting sources (healthcare professionals or non-healthcare professionals). Additionally, we analyzed the annual reporting trend, the most frequently reported suspected ADRs, and the indications for use associated with potentially counterfeit semaglutide-based products.

### 2.4 Disproportionality analysis

We performed a disproportionality analysis by computing the Reporting Odds Ratio (ROR) and its 95% confidence interval (95% CI) to assess if potential counterfeit semaglutide has a different probability of reporting ICSRs with the most frequent suspected ADRs compared with non-counterfeit semaglutide. The most frequent suspected ADRs were defined as those reported in at least 2% of cases. For this analysis, “non-counterfeit semaglutide” was defined as all ICSRs reporting semaglutide as a suspected medicinal product that did not include any of the predefined PTs related to counterfeit or tampering. Crude RORs were calculated without adjustment for potential confounders such as age, sex, or reporting source, in line with standard pharmacovigilance practice.

The *p-value* <0.05 was used for statistical significance. No formal sensitivity analyses were performed to assess the robustness of the findings. All statistical analyses were performed using R Statistical Software (version 4.0.3; R Foundation for Statistical Computing, Wien, Austria).

### 2.5 Ethical consideration

Considering that all ICSRs collected in the EV database are anonymized and do not include any information that could identify the patient, Ethics Committee approval was deemed unnecessary.

## 3 Results

Of the 47,819 reports extracted from EV listing semaglutide as the suspected drug, 0.49% (N = 234 ICSRs) involved a potential counterfeit semaglutide-based product, while the remaining 47,585 ICSRs were classified as non-counterfeit semaglutide (Table 1).

**TABLE 1:** Characteristics of the 234 individual case safety reports (icSRs) including suspected counterfeit semaglutide-based product as suspected drug.

​	Counterfeit semaglutide N. of individual case safety reports (%)	Non-counterfeit semaglutide N. of individual case safety reports (%)
Total	N = 234 (100.0)	N = 47,585 (100.0)
Age category		
0–12 years	0	42 (0.1)
12–17 years	1 (0.4)	112 (0.2)
18–64 years	83 (35.5)	20,619 (43.4)
65–85 years	15 (6.4)	9493 (19.9)
Not specified	135 (57.7)	17,319 (36.4)
Sex		
Female	172 (73.5)	28,052 (59.0)
Male	49 (20.9)	15,892 (33.4)
Not specified	13 (5.6)	3641 (7.6)
Reporting year		
2018	0	191 (0.4)
2019	0	1471 (3.1)
2020	1 (0.4)	2274 (4.8)
2021	1 (0.4)	2903 (6.1)
2022	3 (1.3)	3932 (8.3)
2023	53 (22.6)	6759 (14.2)
2024	84 (35.9)	11,434 (24.0)
2025	92 (39.4)	18,621 (39.1)
Report type = spontaneous	234 (100.0)	47,585 (100.0)
Source qualification		
Non healthcare professionals	134 (57.3)	23,285 (48.9)
Healthcare professionals	100 (42.7)	24,300 (51.1)
Source country		
Non european economic area	197 (84.2)	27,359 (57.5)
European economic area	37 (15.8)	20,226 (42.5)
Median n. of suspected adverse drug reactions (IQR)^a^	4 (3–5)	2 (1–4)

^a^ Median number of suspected drug reaction (IQR) refers to the total number of preferred terms reported per ICSR.

**FIGURE 1:**
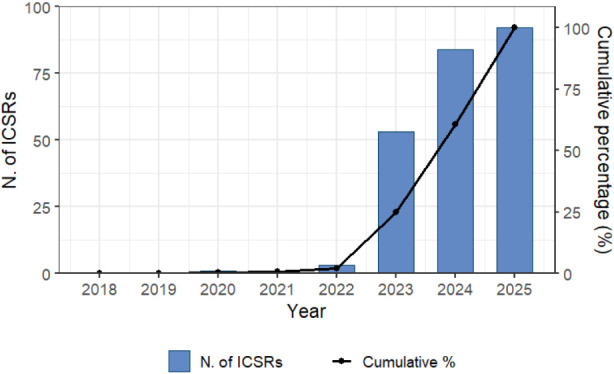
Distribution of Individual Case Safety Reports (ICSRs) having suspected counterfeit semaglutide-based product as suspect drugs by year (2018–2025).

In the counterfeit group, the patient’s age was not reported in most cases (N = 135; 57.7%), compared to 36.4% in the non-counterfeit group. When available, the most represented age category was 18–64 years (35.5% vs. 43.4% in non-counterfeit reports), while patients aged ≥65 years were less frequently reported in counterfeit cases (6.4% vs. 19.9%).Most patients were female in both groups, with a higher proportion observed in counterfeit reports (73.5% vs. 59.0%). All reports were spontaneous (100.0%). Reports involving counterfeit semaglutide were more frequently submitted by non-healthcare professionals (57.3% vs. 48.9%) and more often originated from non-European Economic Area countries (84.2% vs. 57.5%) compared to non-counterfeit reports. Finally, the median number of reported adverse events per ICSR was higher in counterfeit cases than in non-counterfeit cases (4 [IQR 3–5] vs. 2 [IQR 1–4]). As reported in Figure 1, the first reports of potential counterfeit semaglutide products were recorded in 2020, with a peak reached in 2025 (Figure 1).

The majority of ICSRs were classified as serious in both groups, with a higher proportion observed in counterfeit semaglutide compared to non-counterfeit semaglutide (89.3% vs. 66.7%). Among seriousness criteria, *Other Medically Important Condition* was the most frequently reported in both groups, although more commonly in counterfeit cases (63.2% vs. 37.6%). *Caused/Prolonged Hospitalization* was reported in 18.4% of counterfeit ICSRs and 23.8% of non-counterfeit ICSRs. Less frequently selected criteria included *Life Threatening* (3.4% vs. 1.8%), *Results in Death* (3.0% vs. 1.3%), and *Disabling* (1.3% vs. 2.1%) for counterfeit and non-counterfeit semaglutide, respectively. Conversely, non-serious cases were more frequently reported in the non-counterfeit group (33.2% vs. 10.7%) (Table 2).

**TABLE 2:** Seriousness of ICSRs related to semaglutide.

Seriousness	Counterfeit semaglutide N. of individual case safety reports (%)	Non-counterfeit semaglutide N. of individual case safety reports (%)
Total	N = 234 (100.0)	N = 47,585 (100.0)
Serious	209 (89.3)	31,763 (66.7)
Other medically important condition	148 (63.2)	17,914 (37.6)
Caused/Prolonged hospitalization	43 (18.4)	11,348 (23.8)
Life threatening	8 (3.4)	875 (1.8)
Results in death	7 (3.0)	626 (1.3)
Disabling	3 (1.3)	1000 (2.1)
Not serious	25 (10.7)	15,822 (33.2)

Table 3 describes the most frequently reported suspected ADRs for both potential counterfeit and non-counterfeit semaglutide. Overall, gastrointestinal events such as “vomiting” and “nausea” were among the most frequently reported in both groups, although they were more commonly reported in non-counterfeit ICSRs (“vomiting”: 9.3% vs. 13.5%; “nausea”: 8.6% vs. 18.1%).

**TABLE 3:** Frequency of the suspected adverse drug reactions reported in at least 2% of the individual case safety reports with a suspected counterfeit semaglutide-based product.

Suspected ADR (preferred term)	Counterfeit semaglutide N. of individual case safety reports (%)	Non-counterfeit semaglutide N. of individual case safety reports (%)
Total	234 (100.0)	47,585 (100.0)
Vomiting	30 (9.3)	6431 (13.5)
Nausea	28 (8.6)	8623 (18.1)
Hypoglycaemia	24 (7.4)	526 (1.1)
Product use in unapproved indication	23 (7.1)	1584 (3.3)
Off label use	21 (6.5)	4655 (9.8)
Diarrhoea	16 (4.9)	5071 (10.6)
Drug ineffective	15 (4.6)	907 (1.9)
Malaise	15 (4.6)	900 (1.9)
Weight increased	12 (3.7)	809 (1.7)
Blood glucose increased	11 (3.4)	1266 (2.7)
Dehydration	11 (3.4)	1598 (3.4)
Pancreatitis	11 (3.4)	3042 (6.4)
Prescription drug used without a prescription	11 (3.4)	123 (0.3)
Hyperhidrosis	9 (2.8)	238 (0.5)
Tremor	9 (2.8)	210 (0.4)
Abdominal pain	8 (2.5)	4471 (9.4)
Illness	8 (2.5)	423 (0.9)
Loss of consciousness	8 (2.5)	354 (0.7)
Constipation	7 (2.2)	2978 (6.3)
Dizziness	7 (2.2)	1567 (3.3)
Feeling abnormal	7 (2.2)	262 (0.6)
Syncope	7 (2.2)	304 (0.6)
Weight loss poor	7 (2.2)	522 (1.1)
Wrong technique in product usage process	7 (2.2)	961 (2.0)

Conversely, some suspected ADRs were more frequently reported in counterfeit cases, including “hypoglycaemia” (7.4% vs. 1.1%), “product use in unapproved indication” (7.1% vs. 3.3%), “drug ineffective” (4.6% vs. 1.9%), “malaise” (4.6% vs. 1.9%), and “weight increased” (3.7% vs. 1.7%).

Other events such as “off label use” (6.5% vs. 9.8%), “diarrhoea” (4.9% vs. 10.6%), “pancreatitis” (3.4% vs. 6.4%), and “abdominal pain” (2.5% vs. 9.4%) were more frequently reported in the non-counterfeit group.

**FIGURE 2:**
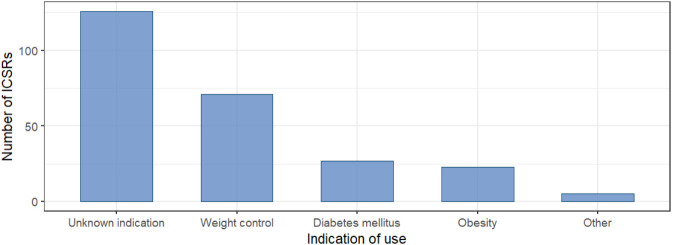
Indication of use of the suspected counterfeit semaglutide-based products included in the individual case safety reports.

In most cases, the indication for semaglutide was not reported (N = 126; 50.0%). Among the cases with available information, semaglutide was most frequently used for weight control or obesity (N = 94; 37.3%), followed by diabetes mellitus (N = 27; 10.7%) (Figure 2).

Looking at the probability of reporting clinically suspected ADR, potential counterfeit semaglutide was associated with a higher risk of reporting “hypoglycemia” (ROR = 10.27, 95% CI 6.38–15.87, *p* << 0.05), “product use in unapproved indication” (not necessarily indicative of an off label use) (ROR = 3.18, 95% CI 1.97–4.92, *p* < 0.05), “malaise” (ROR = 3.57, 95% CI 1.96–6.05, *p* << 0.005), and “drug ineffective” (ROR = 3.54, 95% CI 1.94–6.00, *p* << 0.005) compared to non-counterfeit semaglutide. No statistically significant associations were found for other events (Table 4).

**TABLE 4:** Reporting odds ratio (ror) with 95% confidence Interval (95% CI) related to the suspetected adverse drug reactions (ADRs) associated with potential counterfeit semaglutide-based products versus those associated with non-counterfeit semaglutide-based products. Only suspected ADRs reported in ≥4% of cases are shown.

Suspected ADR (preferred term)	ROR (95% CI)	p
Vomiting	0.95 (0.62–1.39)	0.85
Nausea	0.62 (0.40–0.92)	0.02
Hypoglycaemia	10.27 (6.38–15.87)	<0.05
Product use in unapproved indication	3.18 (1.97–4.92)	<0.05
Off label use	0.91 (0.55–1.43)	0.82
Diarrhea	0.62 (0.35–1.03)	0.07
Drug ineffective	3.54 (1.94–6.00)	<0.05
Malaise	3.57 (1.96–6.05)	<0.05

## 4 Discussion

To our knowledge, no study has investigated the suspected adverse drug reactions (ADRs) associated with the use of potential counterfeit semaglutide products through the analysis of ICSRs collected in pharmacovigilance databases. In this study, we aimed to describe the reported suspected ADRs related to potential counterfeit semaglutide through the analysis of data from the EudraVigilance database. The choice to evaluate these specific safety concerns was prompted by warnings issued by Regulatory Authorities regarding the marketing of counterfeit semaglutide.

Our analysis showed that most analyzed ICSRs (73.5%) were associated with female patients. Literature data suggest that women are larger consumers of semaglutide, especially when the drug is used off-label for aesthetic purposes, particularly for body weight control (Allison et al., 2021). Furthermore, it is also known that women are more prone to suspected ADRs compared to men (di Mauro et al., 2019). The causes of this greater susceptibility include differences in pharmacokinetic, immunological, and hormonal factors, as well as differences in drug use by women compared to men (Rademaker, 2001). In agreement with previous pharmacovigilance studies, most of the ICSRs related to semaglutide concerned patients aged 18–64 years (Du et al., 2024; Nakhla et al., 2024; Butuca et al., 2024). Adult patients show a higher prevalence of overweight and obesity and therefore represent the main consumers of semaglutide (Mayer and Fontelo, 2024; GBD, 2021 Adult BMI Collaborators, 2025). Moreover, the only ICSR collected by EV regarding a patient aged between 12 and 17 years should serve as a warning against the use of such counterfeit products among adolescents, an age group in formation and development, and particularly sensitive to the stigma of being overweight or obese.

All ICSRs were spontaneous (100.0%) and reported by non-healthcare professionals (57.3%). This result differs from what is generally observed in pharmacovigilance studies, where healthcare professionals are usually the most common source of reporting (Rafaniello et al., 2023). On one hand, this result could suggest an increase in interventions to stimulate spontaneous reporting of suspected adverse drug reactions by consumers (patients/citizens) (Rafaniello et al., 2023; Mascolo et al., 2023). On the other hand, it could shine a spotlight on the widespread use of semaglutide-based products outside the official clinical setting and medical supervision.

In terms of the primary source country, almost all cases came from the non-European Economic Area (84.2%), suggesting that most potential counterfeit products are used more in some geographical areas than in others. This result agrees with the warning issued by the WHO (WHO, 2025). Indeed, most counterfeit products have been detected in Brazil, the United Kingdom, Northern Ireland, and the United States of America. In particular, the alert notice concerns 3 counterfeit batches of the drug semaglutide detected in Brazil in October 2023, in the United Kingdom also in October 2023, and in the United States in December 2023 (WHO, 2025; UK Government, 2025). Our analysis showed an increase in ICSRs from 2023 to 2025, with a peak in the last year. In this regard, Regulatory Authorities have increased monitoring activities starting from 2023, raising awareness among citizens and healthcare operators about reporting suspected ADRs from counterfeit semaglutide (European Medicines Agency, 2015). Furthermore, the year 2024 represented a moment of crisis between supply and demand, making semaglutide one of the most requested, counterfeited, and consumed drugs. This could therefore also explain the increase in the number of ICSRs.

It is important to note that several differences observed between counterfeit and non-counterfeit semaglutide reports, including reporter type, seriousness, geographic origin, and the proportion of missing data, may have influenced the observed reporting patterns. These factors are known to affect the probability of reporting specific adverse events in spontaneous reporting systems and, therefore, may act as potential confounders in disproportionality analyses.

Our analysis showed that 89.3% of ICSRs were serious. This finding is not surprising considering the predominance of non-healthcare professionals as the primary source (57.3% of ICSRs were reported by non-HCPs). In line with our data, Avery et al. and Rolfes et al. demonstrated that consumers/patients tend to report suspected ADRs more frequently when these are serious (Avery et al., 2011; Rolfes et al., 2015). However, this proportion should be interpreted with caution, as spontaneous reporting systems are affected by reporting bias, with serious events more likely to be reported. In addition, media attention and regulatory warnings on counterfeit semaglutide may have contributed to stimulated reporting. Notably, the lower proportion of serious reports observed in the non-counterfeit comparator group (66.7%) suggests that these differences may reflect reporting dynamics and potential confounding factors rather than true differences in clinical severity.

The most reported suspected ADRs in suspected counterfeiting cases were “vomiting”, “nausea”, “hypoglycemia”, “diarrhoea”, “malaise”, “dehydration”, and “pancreatitis”. GLP-1 receptor agonists are generally well-tolerated but also associated with these side effects, already reported in the Summary of Product Characteristics (European medicines agency (EMA). Ozempic, 2025). For example, the main peripheral cause of nausea is the effect of semaglutide on the gastrointestinal tract; delayed gastric emptying, although useful because it prolongs satiety and reduces nutrient absorption, can cause nausea and vomiting. However, semaglutide can also trigger such events through a direct signaling pathway in the central nervous system (Smits and Van Raalte, 2021; Chaudhry et al., 2024). The frequency of the events described above has also been highlighted in recent pharmacovigilance studies (Du et al., 2024; Liu et al., 2022). These suspected ADRs are usually mild to moderate in severity, of short duration, and tend to improve over time (Amaro et al., 2022). According to the study conducted by Liu and colleagues, semaglutide showed a higher risk of nausea, diarrhea, and vomiting compared to all other GLP-1 RAs (Liu et al., 2022). Although several studies have reported cases of hypoglycemia associated with the use of GLP-1RAs, the main evidence available comes from clinical trials (Rolfes et al., 2015).

Among the most serious cases, pancreatitis emerged from our analysis as a suspected ADR related to the use of potential counterfeit semaglutide products. Literature data suggest that treatment with GLP-1 RAs is associated with an increased incidence of pancreatitis (Chis and Fodor, 1957; Li et al., 2014; Faillie et al., 2014). A pharmacovigilance study observed that the number of cases of acute pancreatitis steadily increased from 16 in 2005 to 459 in 2023, reflecting the growing clinical use of GLP-1 RAs (Guo et al., 2024). The mechanism underlying the onset of pancreatitis with semaglutide is not yet fully understood, but several hypotheses have been made, supported by preclinical and clinical studies (Hughes et al., 2024; Butler et al., 2013). Among them, there is the direct activation of GLP-1 receptors on pancreatic cells, which causes an excessive growth of the cells that line the smaller ducts and subsequently causes inflammation (Butler et al., 2013; Ryder, 2013). Generally, semaglutide-induced pancreatitis may be underestimated because patients with diabetes often present other risk factors, such as obesity, and are treated with other medications that may increase the risk of pancreatitis (Dagher et al., 2024).

Although the therapeutic indication was not reported in half of the ICSRs, 37.3% indicated that semaglutide was used for weight control or obesity, highlighting that in most cases the potentially counterfeit product was used for its weight loss effect rather than for the treatment of diabetes mellitus, underscoring the growing interest in their use (Gasoyan et al., 2025). Finally, our study showed an increased probability of reporting “hypoglycemia”, “product use in unapproved indication”, “malaise”, and “drug ineffective” with potential counterfeit semaglutide compared to non-counterfeit semaglutide. Regarding “drug ineffective”, this result is not surprising, as according to the literature, counterfeit drugs may not meet the same standards of quality, safety, and efficacy compared to the original (Ozempic Semaglutide and Saxenda Liraglutide, 2026). Furthermore, Gasoyan et al. have demonstrated that the effectiveness of GLP-1 drugs in real-life is closely related to two aspects: the continuity of care and adherence to the correct dosage; when one of the two is missing, effectiveness is reduced (Hach et al., 2024). Finally, it cannot be ruled out that the use of this drug without the clinician’s supervision for the most appropriate dosage (characterized by an up-titration to the maximum tolerated maintenance dose, which can differ depending on the indication for glycemic control or overweight and obesity) could contribute to drug ineffectiveness. The higher reporting of hypoglycemia may be related to the presence of insulin or other potentially toxic substances in counterfeit semaglutide products as reported by Regulatory Authorities (World Health Organization and the EMA) (NABP, 2023; Food and Drug Administration, 2025; Ozempic Semaglutide and Saxenda Liraglutide, 2026; Hach et al., 2024). Our analysis also highlighted the presence of several PTs suggestive of a lack of effectiveness. In our opinion, the lack of effectiveness could be due to the absence of the active ingredients approved by the FDA or EMA, or to non-compliant dosages, as reported in some cases documented by the Regulatory Authorities (Liguori et al., 2023).

The higher likelihood of reporting “product use in an approved indication” with counterfeit semaglutide could further highlight how the back market has contributed to increased sales of semaglutide-based products for individuals not eligible for this treatment (e.g., adults with an Body Mass Index of <27 kg/m^2^ and in the absence of at least one weight-related comorbidity). However, the Preferred Term “product use in unapproved indication” does not necessarily imply inappropriate use, and may also reflect cases in which a counterfeit product was used within a clinically appropriate indication. Finally, the suspected ADR “malaise”, classified under the System Organ Class (SOC) “General disorders and administration site conditions” in MedDRA, is not present in the Summaries of Product Characteristics (SmPC) of both medicinal products containing semaglutide and authorized by the EMA and FDA (Wegovy® and Ozempic®). Malaise is a common but complex symptom that can indicate a wide range of underlying health problems (e.g., dizziness, headache, nausea, fatigue), to which users of counterfeit drugs (including semaglutide) unknowingly or carelessly expose themselves.

## 5 Strengths and limitations

This study has several strengths. EudraVigilance represents one of the largest databases of spontaneous reports, collecting information from various countries. In addition, it indirectly allows for the identification of potential cases of semaglutide counterfeiting.

Generally, pharmacovigilance databases represent a fast and cost-effective method for identifying a possible association between a drug and a suspected adverse drug reaction (ADR), to identify a new ADR, including rare and unexpected reactions that cannot be identified during the pre-marketing phase (Liguori et al., 2023; Mascolo et al., 2025). However, the spontaneous reporting system is characterized by intrinsic limitations mainly related to underreporting and the inaccuracies or incompleteness of information. In this regard, important information may not have been reported in the ICSRs we analyzed (e.g., concomitant conditions, other additional suspected or concomitant drugs). Moreover, EudraVigilance contains exclusively data on the safety of medicines. Indeed, there is a lack of information on the number of patients treated with potential counterfeit semaglutide. For this reason, it is not possible to calculate incidences or other risk measures, but it can provide an overview of cases involving potentially counterfeit semaglutide and support the identification of potential new safety signals. Another important limitation concerns the identification of counterfeit cases. Some reports classified as counterfeit may represent suspected rather than confirmed falsified products, while other counterfeit exposures may not have been recognized or reported as such. In particular, incidents involving counterfeit medicines are often underreported due to lack of awareness, fear of legal consequences, or difficulties in reporting suspicions, potentially leading to an underestimation of such cases. At the same time, reporting may also be influenced by differential reporting behaviors, as patients or consumers could be more likely to report adverse events when a counterfeit product is suspected, introducing a potential reporting bias between counterfeit and non-counterfeit cases (Pozsgai et al., 2022; Raj et al., 2025). This potential misclassification of exposure could have biased the results in either direction. Furthermore, EudraVigilance does not provide reliable information on the actual composition, dose, or quality of counterfeit products, preventing a more detailed assessment of the relationship between product characteristics and reported suspected ADRs. Moreover, due to the observational nature of pharmacovigilance data, no causal relationship between exposure and suspected ADRs can be established, and the findings should be interpreted as reporting associations rather than evidence of causality. Finally, the relatively small number of ICSRs related to potential counterfeit semaglutide may limit the statistical precision of some analyses. Therefore, the results should be interpreted with caution, particularly for suspected ADRs based on a limited number of reports.

## 6 Conclusion

This pharmacovigilance study provides a characterization of suspected adverse drug reactions (ADRs) associated with potential counterfeit semaglutide using data from the EudraVigilance database. Our findings show that reports involving potential counterfeit semaglutide were more frequently associated with serious cases and a higher number of reported suspected ADRs compared to non-counterfeit semaglutide. The most commonly reported suspected ADRs were consistent with the known safety profile of GLP-1 receptor agonists; however, disproportionality analysis highlighted a higher reporting probability for specific events, including hypoglycemia, drug ineffective, malaise, and product use in unapproved indication in counterfeit cases.

These findings suggest that counterfeit semaglutide may be associated with distinct reporting patterns, potentially reflecting differences in product quality, inappropriate use, or lack of therapeutic efficacy. However, given the inherent limitations of spontaneous reporting systems and the potential influence of confounding factors, these results should be interpreted with caution.

Overall, our study highlights the value of pharmacovigilance systems in detecting potential safety signals related to counterfeit medicines and supports the need for continued monitoring and further research in this area.

## Data Availability

The raw data supporting the conclusions of this article will be made available by the authors, without undue reservation.
